# Development of a Tool to Increase Physical Activity among People at Risk for Diabetes in Low-Resourced Communities in Cape Town

**DOI:** 10.3390/ijerph17030865

**Published:** 2020-01-30

**Authors:** Jillian Hill, Camille Lavigne Delville, Anne-Marie Auorousseau, Deborah Jonathan, Nasheeta Peer, Brian Oldenburg, Andre-Pascal Kengne

**Affiliations:** 1 Non-Communicable Diseases Research Unit, South African Medical Research Council, Cape Town 7505, South Africa; Deborah.Jonathan@mrc.ac.za (D.J.); nasheeta.peer@mrc.ac.za (N.P.); andre.kengne@mrc.ac.za (A.-P.K.); 2 Bordeaux School of Public Health, University of Bordeaux, Bordeaux, 33100 Nouvelle-Aquitaine, France; camille_ld@orange.fr (C.L.D.); a-marie.aurousseau@hotmail.fr (A.-M.A.); 3 Melbourne School of Population and Global Health, University of Melbourne, Melbourne, VIC 3010, Australia; brian.oldenburg@unimelb.edu.au

**Keywords:** diabetes prevention, physical activity, intervention tool, low-resourced communities

## Abstract

Targeted lifestyle interventions, including physical activity (PA), have been proven to prevent or delay the onset of diabetes. South Africa’s unique context, complex environment and varied cultures and ethnicities require tailored interventions. Our objective was to develop a context-appropriate tool for the South African Diabetes Prevention Programme’s PA lifestyle component in order to enable people at risk of developing diabetes to adopt PA. We used mixed methods to inform the development of the tool. Descriptive analyses of baseline survey data included socio-demographics, anthropometrics, blood pressure and biochemical measurements, reported medical history, PA behaviours, and built environment information. Focus group discussions assisted in understanding perceived challenges, barriers and facilitators/opportunities to PA. A literature search on successful South African PA interventions was done, and PA experts in Cape Town were consulted. Quantitative data were analysed using the software R, version 3.4.4 and qualitative data were thematically analysed. Participants (n = 316) recruited were mostly black (54.4%) and of mixed-ancestry (44.6%); they were mainly female (80.1%), obese (75.2%), and had an haemoglobin A1c (HbA1c) above 5.7% (65.5%), with 30% having hypertension and 87% (self-reported) meeting the World Health Organisation (WHO) PA recommendation. Main barriers to PA practice were safety, cost and accessibility of sports facilities, and laziness. We included practising moderate-intensity aerobic and resistance exercises and take-home self-help materials as recommended. By combining results, we produced a targeted, practical and promotional PA booklet.

## 1. Introduction

Non-communicable diseases (NCDs), including type 2 diabetes (T2DM) are on the rise globally. In South Africa (SA), the prevalence of T2DM, at 5.5%, is the second highest in Africa according to the International Diabetes Federation (IDF) [1]. This national figure likely conceals major subnational (provincial) as well as ethnic differences in the diabetes burden that remain to be fully characterised. South African burden of disease research has shown that the prevalence of diabetes has nearly doubled from the year 2000 to 2009, from an estimated 5.5% [1] to that of 9% [2]. An update on diabetes in sub-Saharan Africa showed that diabetes in South Africa was highest among Indians (13%), followed by people from mixed-ancestry (10.8%) then black Africans (5.3%) [3]. Repeated surveys in the province of the Western Cape, South Africa, have shown that the prevalence of diabetes increased from 8.0% in 1990 to 12.2% in 2008 in black Africans in Cape Town [4], while the age-standardised prevalence rate in 2008/09 was 26.3% in mixed-ancestry South Africans residing in the same city [5]. These figures support diabetes prevalence rates higher than the national average in Cape Town, including among population groups previously considered to be at low risk of diabetes.

The burden of T2DM and NCD is largely influenced by the economic development of a country, the change in lifestyle it enables, and an ageing population. Factors include urbanisation, nutrition and economic transitions, smoking, alcohol consumption and a decrease in physical activity (PA), coupled with increased sedentary behaviour [1,3,4].

Evidence from studies have shown that increased PA (including type and intensity) and lifestyle changes are effective in reducing the incidence of T2DM and other NCDs [5,6,7,8,9]. Targeted lifestyle interventions, such as the United States Diabetes Prevention Programme and the Finnish Diabetes Prevention Study, have proven that the onset of T2DM can be prevented or delayed in those at risk of developing the condition by up to 58% [10,11].

The South African Diabetes Prevention Programme (SA-DPP) is a cluster randomised controlled trial in its initial phases. The overall aim is to develop and evaluate a culturally relevant model for T2DM prevention in South Africa and is based principally on the Finnish Good Ageing in Lahti Region Lifestyle “real world” Implementation Trial [12]. The lifestyle change objectives (diet and PA) of the current programme are the same as those in the original Finnish Diabetes Prevention Study, i.e., (1) <30% of total energy intake from fat; (2) <10% of total energy intake from saturated fat; (3) >15 g of fibre/1000 kcal; (4) >4 h/week moderate level of physical activity; and (5) >5% body mass index (BMI) reduction [10]. The SA-DPP target populations are black and mixed-ancestry individuals (25–65 years old) at risk of developing T2DM residing in low-income communities in Cape Town. The pilot recruitment phase was used to adapt existing DPP lifestyle intervention curricula to be suitable for implementation in black African and mixed-ancestry populations from low-resourced communities. The opportunity to develop targeted complimentary tools (for nutrition and PA) arose. 

The objective of this paper is to present the process followed in developing a suitable PA tool to promote increased PA in resource-poor communities at risk of developing T2DM.

## 2. Methods

A mixed-method approach (using quantitative and qualitative methods) which comprised four steps was used to develop the PA tool (booklet). 

### 2.1. Step 1: A Descriptive Analysis of Existing SA-DPP Pilot-Phase Baseline Screening Data Was Done. The Main Objective Was to Identify the Socio-Demographic Profile as Well as Health-Risk Profiles of People at Risk of Developing Diabetes

*Data collection:* SA-DPP participants were recruited between August 2017 and March 2018. Black and mixed-ancestry participants, of ages ≥25–65 years, without known T2DM, from eight low-socioeconomic communities (formal and informal townships) were screened using a brief questionnaire, and anthropometric and blood pressure (BP) measurements to estimate their risk of T2DM by using the African Diabetes Risk Score (ADRS). The ADRS considers age, waist, BP and has slightly different cut-off points for black and mixed-ancestry participants. An oral glucose tolerance test, other biochemical and clinical assessments, and a detailed questionnaire (ten sections: general information, socio-demographic characteristics, health, medication, family medical history, alcohol use, tobacco use, PA information, dietary history and support networks) were thereafter administered to participants identified at high risk for T2DM. The following exclusion criteria was applied: being younger than 25 or older than 65 years of age, having existing diabetes, being bedridden or breastfeeding, or having been diagnosed with tuberculosis or cancer in the past six months. Informed written consent was obtained from all participants. 

*Definitions:* In this study, elevated haemoglobin A1c (HbA1c) was defined as ≥5.7 mmol/L. Hypertension was defined as BP ≥140/90 mmHg or being previously diagnosed by a doctor or nurse. High normal blood pressure was defined as 130 to 139 mmHg systolic and/or 85 to 89 mmHg diastolic. BMI was computed as kg/m^2^. Overweight (not obese) if BMI is 25.0 to 29.9, class 1, (low-risk) obesity, if BMI is 30.0 to 34.9, class 2 (moderate-risk) obesity, if BMI is 35.0 to 39.9 and class 3 (high-risk) obesity, if BMI is equal to or greater than 40.0. Chronic stress was defined as experiencing a stressor continuously for the past 12 months (e.g., health problems (self or in a close relative or friend), difficulties with a job or ability to work, financial strain or difficulty in a close relationship [13]).

*Measures:* The variables selected for analysis as part of this objective were Socio-demographic data, i.e., age, gender, area, marital status, education level, occupation and household income; Behavioural measures, i.e., tobacco and alcohol use as well as sleep; Psychological measures, i.e., chronic stress; PA measures, i.e., PA pattern and barriers to PA; medical history, i.e., family history of T2DM; clinical measures, i.e., waist, weight, height, BP and glycated haemoglobin (HbA1c); Neighbourhood living conditions indicators, i.e., stores and facilities in the neighbourhood, access to services and places, roads and walking paths in the neighbourhood, places for walking, cycling and playing, neighbourhood surroundings, safety from traffic, safety from crime, personal safety, and child-related questions on stranger danger (see Table 1).

*Data analysis*: The required data were downloaded from the electronic database. A database was then created and cleaned in Excel. Data coherence, validity, reliability, and exploitability were checked. For the global physical activity questionnaire (GPAQ) variables, particular controls and coding are prescribed [14]. The methods were strictly followed. The Neighbourhood Environment Walkability Scale (NEWS) Africa required a scoring methodology to analyse the results [17], methods that have been strictly followed.

Descriptive analysis was conducted with the software R, version 3.4.4. Results are expressed as median and 25^th^–75^th^ percentiles or mean and standard deviation (SD) for quantitative variables and counts and percentages for categorical variables. 

### 2.2. Step 2: Focus Group Discussions (FGDs) Were Conducted to Identify the Barriers and Facilitators to PA That Participants Experienced

*Data collection:* SA-DPP participants from four out of the eight areas (first four that reached sufficient numbers for study inclusion) who were identified as at risk of developing diabetes were approached to participate in FGDs. A semi-structured interview schedule was developed, which was informed using a combination of literature [18,19] and consultation with SA-DPP project team members. Seven open-ended questions were included covering various aspects of PA, i.e., (1) own understanding/concept of PA; (2) PA practice (type, condition and interest); (3) source of motivation/PA facilitators; (4) social support; (5) barriers to PA; (6) opinion and (7) need for the development of a PA tool to support/increase PA. After the first FGD, engagement with the analysis of descriptive results (Step 1) and discussions post-FGD, two additional questions were added. These were (1) self-efficacy regarding PA, and (2) tools or resources currently used to obtain exercise ideas. FGDs were conducted in English, isiXhosa or Afrikaans, with the use of a translator when required. The project manager, who was experienced in qualitative data collection, conducted or supervised the FGDs. All the FGDs were audio-recorded, with written informed consent obtained from participants, and transcribed verbatim.

*Data analysis*: A thematic analysis approach [20] was followed using a stepwise approach. First, a meeting with key project team members was held to consider the approach and the pre-determined themes that guided the FGDs emerged from these. Two independent coders developed an initial code list based on the two initial FGDs using open coding (i.e., allocating codes and giving them a concise label) [21]. The code list was then compared, and agreement reached in conjunction with the project manager. As the coding continued, the code list was refined by reviewing, merging, deleting and adding codes where applicable, which was done a few times. The final code list is presented in Appendix A. 

### 2.3. Step 3: Comprised a Review of the Literature

*Data collection:* A systematic approach was applied to identify previous programmes aimed at promoting healthy lifestyles among low-income communities in South Africa. National and international guidelines were also searched to further inform the development of the tool. 

PubMed, Scopus and Google Scholar were searched using the Medical Subject Headins (MeSH) equation “(((((“PA” [Title/Abstract] OR “exercise” [Title/Abstract] OR “exercise” [MeSH Terms] OR “physical fitness” [Title/Abstract] OR “behavior*” [Title/Abstract] OR “lifestyle” [Title/Abstract] OR “exercise therapy” [MeSH Terms] OR “physical fitness” [MeSH Terms] OR “life style” [MeSH Terms] OR “health behavior” [MeSH Terms])) AND (“education*” [Title/Abstract] OR “intervention*” [Title/Abstract] OR “program*” [Title/Abstract] OR “prevention” [Title/Abstract] OR “promotion” [Title/Abstract] OR “physical education and training” [MeSH Terms] OR “education” [MeSH Terms])) AND (“SA” [Title/Abstract] OR “SA” [MeSH Terms])) AND (“2005/01/01” [PDat]: “2018/12/31” [PDat]))”.

*Data analysis (extraction and synthesis)*: Information from articles was extracted into a reading grid which contained the authors’ name/s, the year of publication, the main objective of the study, the study location and its design, information about participants of the study (number, characteristics and age), the intervention details and the principal outcomes. (The complete strategy and results—Appendix B).

### 2.4. Step 4: Comprised Expert Consultations (PA Experts with Experience in Working with Low-Income/Resourced Communities) and Engagement with the SA-DPP Project Team. Discussions Focused on Appropriate Tools, Exercises and Appropriate Intervention/Educational Content

The information derived from the step-wise mixed-data collection methods and subsequent data integration, assisted the researchers together with the experts in developing a clear understanding of the key elements, barriers and facilitators to PA and its relevance to the target population, i.e., people in low-resourced communities at risk of developing diabetes. Thus, this enabled the development of a contextually appropriate PA tool to promote and increase PA in the above-mentioned population.

Ethical clearance was obtained from the Ethics Committee of the South African Medical Research Council (approval no. EC018-7/2015).

## 3. Results

### 3.1. Socio-Demographic and Health Profile of the Study Population 

A total of 316 participants at risk of developing T2DM from eight townships in Cape Town with valid information for the selected variables were included in the analysis. Participants had a median age of 53 years (25th–75th percentiles = [46–59]), with 18% younger than 45 years of age and 81% older. They were mostly female (80.1%), and the distribution of black and mixed-ancestry was relatively similar—54.4% and 44.6%, respectively. These participants had low levels of education and income and were typically unemployed. Sixty-eight per cent had never reached a level above that of high school, 75.6% earned less than R3 200 (± 220$) per month for the entire household, and 38.9% were unemployed (Table 2).

In Table 3, the main behavioural and biological risks factors for T2DM in the population are summarised. The median waist circumference and BMI of the sample was 102 cm (IQR = [95.0–111.11] and 34.85 kg/m^2^, (IQR = [29.93–40.74]), respectively. Regarding BMI, 73.7% of the participants were obese, with 39.1% of them classified as high-risk obesity (class 3), being morbidly obese. The genetic risk factors were present, with almost half (44.6%) of the participants known to have a close relative (mother, father, sister or brother) affected by diabetes. Though many participants had an optimal or normal BP (40.8%), 22.5% had a high normal one, and 29.4% were already suffering from hypertension. Nearly two-thirds (65.2%) had an HbA1c above 5.7%, which the SA-DPP defines as an individual at-risk of developing T2DM.

Regarding their lifestyles, participants were not heavy consumers of alcohol, with 13.3% drinking alcohol every week (≥1 drink). The consumption of tobacco was much higher; 26.3% of participants identified as current smokers, and nearly half of these (48.7%) reported being chronically under stress. 

### 3.2. Self-Reported Physical Activity Practices 

Self-reported PA pattern among participants is summarised in Table 4. The median duration of PA was approximately 720 min per week (25th–75th percentiles = [240.00–1710.00]), with 86.9% of participants meeting the World Health Organisation (WHO) PA recommendations (150 min of moderate activity or 75 min of vigorous activity per week). The median duration of sedentary behaviour was almost double that of PA, reaching 1260 min per week (25th–75th percentiles = [630.00–1680.00]). 

Looking at the duration of PA per week, a median of 360 min (25th–75th percentiles = [0.00–1050.00]) was completed at work, i.e., for paid or unpaid activities, 180 min (25th–75th percentiles = [60.00–360.00]) for transport, and 30 min (25th–75th percentiles = [0.00–180.00]) for leisure-time activities. Most participants reported performing PA, with 95.1% undertaking PA in general, 72.5% at work, 86.9% for transport and 51.5% during leisure-time. 

### 3.3. Self-Reported PA Aptitude and Barriers 

**Figure 1:**
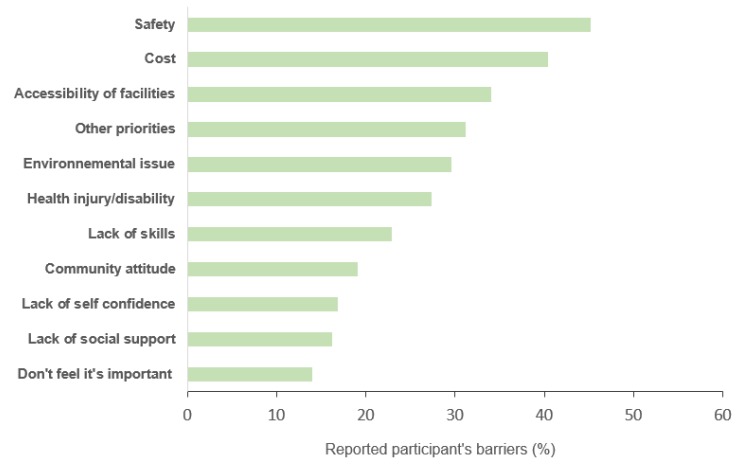
Perceived barriers preventing physical activity participation among at-risk type 2 diabetes black and mixed-ancestry communities, Cape Town, 2018 (N = 316, NA = 2).

The reported barriers (baseline questionnaire) preventing the participants from undertaking PA are shown in Figure 1. The three main barriers were issues of safety (45.2%), the cost of accessing sports facilities (40.4%) and the accessibility of these same facilities (34.1%). Other factors perceived as a barrier by less than one-third of the participants were reported as other priorities (such as work, family needs) (31.2%), environmental issues (like rainy season, pollution) (29.6%), health injury (27.4%%), lack of PA skills (22.9%), community attitude (19.1%), lack of self-confidence (16.9%), lack of social support (16.2%), and the feeling that sport is not important (14.0%). 

Aptitude challenges reported by the study population are presented in Table 5. Participants’ health status did not appear to place a significant limit to do their daily basic activities. Less than 30% perceived their health status limited their ability to walk (23.3%–26.1%), or to climb one flight stairs (23.6%) and lifting groceries (19.7%). Participants encountered more difficulties to bend/kneel (31.5%), to do moderate activities (32.2%), or to climb several stairs (33.1%). Almost two-thirds (64.3%) declared that their health status could be a limit for doing vigorous-intensity activities.

### 3.4. Perceived Neighbourhood and Environment (NEWS Africa Questionnaire)

The highest scores computed for the NEWS scales were access to services and places, roads and walking paths, and personal safety subscales, which reached a median of 3.14 (25th–75th percentiles = [2.71–3.86]), 3.40 (25th–75th percentiles = [2.60–3.40]), and 3.00 (25th–75th percentiles = [3.00–4.00]), respectively. These scores revealed easy walkability linked to good accessibility to non-residential uses (i.e., shops, community facilities), effective street connectivity (“… measures the density of networks and directness of paths” [22]: (1) and a good area vibe (being able to talk to people on the street and not fear too many stray dogs). 

The median scores for the following were average: subscale destinations score (2.62, 25th–75th percentiles = [2.19−3.05]), recreation score (2.50, 25th–75th percentiles = [1.50−3.50]), places for walking, cycling, and playing (2.50, 25th–75th percentiles = [2.00−3.00]), and neighbourhood surroundings (2.50, 25th–75th percentiles = [1.78–3.25]). The proximity to non-residential land uses, the existence of infrastructures for walking and cycling, and the neighbourhood aesthetics were time parameters, indicating medium walkability. 

Inversely, the lowest NEWS-derived scores were the traffic safety, crime safety, and low stranger danger subscales, with scores decreasing until 1.80 (25th–75th percentiles = [1.60–2.60]), 1.25 (IQR = [1.00–2.25]), and 1.00 (25th–75th percentiles = [1.00–1.50]) on average, respectively. Very poor walkability was attributable to issues of safety, crime and traffic reasons, and particularly, true for parents (concern for children).

### 3.5. Qualitative (FGD) Data

A total of seven FGDs were conducted, which included 68 participants from four communities. Participants were mostly female [n = 50], between ages 45 and 65 years [n = 58], and predominantly black [n = 42] rather than mixed-ancestry [n = 26] participants. 

#### 3.5.1. Types of Activities Engaged in by Participants 

The types of activities engaged in daily tended to be of light to moderate intensity. These mostly included household chores (cooking, cleaning, washing clothes) and running errands (such as shopping). Activities also included neighbourhood watch (walking about/patrolling the streets), voluntary work at the retirement home and street cleaning.

*My activity is cleaning the house. Up and down the stairs with the washing because I live in a flat. And walking to Athlone when I go shopping, but I come back with a taxi because of the stuff*.

[Area D, participant 7]

Walking as a means of transport, or as recreation/leisure, appeared to be a vital activity for most participants to keep active. This means of transportation was described as walking to the mall, church, to visit friends, as well as walking to the public transport halt or walking to work.

*Monday to Friday I walk to school to sell snacks to the school children every day so, I do walking.*

[Area B, participant 6]

Some participants walked alone. For others, it was an opportunity to join a group of peers. Walking allowed participants to feel fit or to relax and socialise. Other activities mentioned by participants were sit-ups, push-ups, weight lifting, stretches, (following) television exercises, aerobic exercises (group Zumba/community groups) and skipping rope.

*For us it is more like walking because we don’t go to the gym and pick up weights and stuff. We just do the walking in the morning or in the afternoon or wherever we go. We don’t actually take a car or a bus, we try to actually walk to try and get to that place.*

[Area D, participant 2]

*My stomach used to be big and I do sit-ups so I see that my stomach becoming flatter.*

[Area B, participant 1]

#### 3.5.2. Facilitators to PA 

Participants across all groups were well aware of the many benefits of engaging in PA. Benefits mentioned during the FGDs included health (“wellness”), body, self-confidence (“feeling good in your body”) and stress relief. PA was also often cited as a means of weight management, either to lose weight or to maintain a healthy weight. Participants seemed to have a positive attitude towards PA and did not perceive it as boring or useless, but rather as being beneficial and enjoyable.

*PA impacts your health. It gives good results meaning that when you do exercise you don’t get sick. To do exercise it means taking the stairs, try to do running or walking and jumping like you see people.*

[Area H, participant 4]

*When you start seeing results with diet and exercise you will get confidence to exercise. I have a large tummy but if I exercise I know it will become flatter. So, if you exercise your weight will go down and you can start wearing the clothes sitting in the wardrobe because you gained weight.*

[Area H, participant 4]

About enablers to PA, social support was deemed important. Most participants in the FGDs were part of a community walking group or club. Others were part of the neighbourhood watches in their community and thus patrolled the streets and saw this as sufficient PA.

*I and other people decided as a group to walk twice a day. We walk early in the morning and we also walk in the afternoon. […] We see changes in the body, some of us in our walking group have lost weight and we can move faster and are more flexible.*

[Area B, participant 4]

*No, but I am a neighbourhood watch with participant no. 3. We do exercise because we walk around the community all the time, we are always running after these bad guys stealing people’s cell phones.*

[Area H, participant 7]

*I am very fit. I wake up at 4 o’clock in the morning to do my work as a neighbourhood watch.*

[Area H, participant 3]

Most participants believed that their friends and family would support them in doing PA.

*“My family would be supportive. Everybody in my household likes exercising and going to the gym. The kids they do their chores and go to the gym afterwards.”*

[Area B, participant 3]

*“They will support me.”*

[Area G, participant 7]

*No, I think they will join, they will enjoy it and we do it together, yes.*

[Area D, participant 5]

*My family and friends would be supportive because even my kids gym.*

[Area G, participant 3]

*Like me and Carol, we like to do exercise and my children love it because I do dancing, I do things, they think I am the grandest granny.*

[Area G, participant 1]

#### 3.5.3. Barriers to PA 

A key barrier identified by participants was laziness and or a lack of motivation.

*There are no barriers we need to be disciplined, we are just lazy to exercise we sit the whole day.*

[Area H, participant 4]

The second most highlighted barrier participants mentioned was the lack of infrastructure or venues available for PA. Few venues are dedicated to PA practice, and if present, they are closed or under-utilised because of the absence of a trainer. The absence of appropriate and safe parks was also mentioned.

*“Yes, there is a park. In the park there is three or four stuff to help you to do some exercise, it is there and the community is safe to do it in. You can walk in your community, no one will disturb you.”*

[Area D, participant 2]

Sometimes participants living in the same community have different perceptions on the availability of infrastructure, by saying facilities are available or not.

*I don’t have any barriers because I work in the hall where people come to exercise so I can exercise at the community hall because the facilities are there and I can also work in my garden.*

[Area B, participant 1]

*There are no places to exercise, no parks.*

[Area B, participant 2]

The perception of safety also appeared to vary from person to person. However, most participants felt that safety was a real concern, with shootings and robberies occurring frequently.

*Even though there is a park close to my community to exercise it is not safe. There are robbers waiting to rob you when you are done.*

[Area B, participant 3]

#### 3.5.4. Tools and Resources Currently Used to Support PA 

The most common tool used by participants to facilitate PA practice was the television, which would consist of watching and following an exercise programme, where a trainer/instructor would demonstrate and motivate people to engage in exercise (usually, aerobic exercise). The advantage of television programmes is that they are accessible and motivational.

*TV is a tool I use. At 6 o’clock every morning there is a programme with a lady and two guys exercising. Me *[sic.]* and my daughter watch and follow the exercise with them. I exercise every day using the TV programme before going to work.*

[Area H, participant 7]

*“Seeing exercise on TV also motivates me because when you are at home you can copy what you see if you can’t join the community groups.”*

[Area H, participant 4]

Participants communicated the need for social support and a source of ideas to increase PA practices.

*I need ideas on the types of exercise. At the old age home, they have a book in the library that shows different exercises. It would be nice if someone can do the exercises practically and teach me more exercise. [...] I want to do more exercising. I want to learn about different types of exercises I can do to my body [...]. If you can give us some pamphlet or booklet in English or isiXhosa to assist us to exercise at home.*

[Area H, participant 5]

#### 3.5.5. Considerations from the Literature for Inclusions in a PA Tool 

A complete description of the literature review is attached in Appendix B. Exercises recommended for T2DM, or its prevention were aerobic exercises [23], resistance training [24,25], flexibility exercises [23,26] and balance exercises [27]. However, only resistance [24,25,28,29] and aerobic exercises [23] have been proven to be effective in T2DM control. Aerobic exercises [30,31,32] and the use of a pedometer during walking [33] showed improvement in body health as well as improved compliance to PA among participants when done correctly. In various studies, low-intensity activities and moderate activities such as aerobic exercise with warm-up, work-out and cool-down stages have been advocated as the most appropriate [30,31,32,33,34,35]. In some studies that implemented high-intensity exercise, the dropout rate was high, or a lot of participants could not meet the recommended level of performance [35,36]. The study by Pillay et al. [35] also highlighted the importance of not only considering the duration of the exercise but also the volume and intensity. Light-intensity PA is not sufficient to prevent T2DM; PA of at least moderate intensity is recommended [26,37,38]. People at risk of developing T2DM should minimise sedentary times. Therefore, it is recommended that they interrupt those sedentary periods with frequent bouts of light- to moderate-intensity activities, such as a little aerobic movement or standing up from a sitting position [26,37,38]. 

Regarding the frequency, most guidelines recommend practising exercise regularly and doing different kinds of exercise in a training programme [23,26,39,40]. Given that increases in insulin sensitivity decline after 48 h post-exercise, it is recommended to not stay without training for two consecutive days [23,40,41].

PA promotion tools such as posters [42], pamphlets [42,43], organised PA events [42], certificates [44], a diary in which participants documented their daily step-count [33] have all been used with variable amounts of success. The importance of the development of suitable tools for implementation during the interventions has been displayed in some studies [42,44]. In one study, targeting T2DM, participants requested self-help materials to take home after the intervention [44]. Posters and pamphlets appeared to be the most effective PA tools, containing creative pictures with captions, which drew a lot of attention and interest from participants to ensure that they did not become bored or lose interest [42]. Researchers have demonstrated that educational materials are generally experienced positively, draw attention/interest, and are frequently used [42,43,45].

## 4. Discussion

### 4.1. Compiling the PA Tool 

The findings from the literature review, descriptive analysis and FGDs, together with discussions with experts, presented various tools, types of exercises, the application thereof, and the related intensities for consideration for inclusion in a PA promoting tool.

The study population was mainly female, obese, had class 3 obesity, a median waist circumference of 102 cm, over one-quarter had hypertension, with far more who had an elevated HbA1c. Most participants were ≤45 years old and had low levels of education. Most of them met the PA WHO recommendations, according to their self-reports (GPAQ). However, time spent sedentary was close to double of that of PA. With the validity of GPAQ results being varied among different population groups in South Africa [6,7], self-reported PA should be interpreted with caution. Main barriers to PA practice were around issues of safety, cost and accessibility of sports facilities, and laziness. Facilitators to PA were recognised as social support and needs included access to equipped facilities (with trainers/instructors) and ideas for exercise. Recommendations from the literature included practising aerobic and resistance exercises with moderate intensities, and self-help materials to take home. 

After having reviewed the possible tools as suggested by the literature and other inputs (experts, FGDs and the SA-DPP project team), weighing up the advantages and disadvantages against the economic and technical resources within the SA-DPP study, developing a PA booklet was deemed plausible. The booklet (Appendix C) includes practical information and exercise programmes. Five aerobic exercise sequences (moderate-intensity) are introduced in the booklet, offering easy at-home exercises that can be done in various contexts. These include the ability to exercise while seated, while watching television, whilst busy in and around the house, with a partner, and without bought/specialised material. These exercise sequences have been supplemented by warning (pre-cautionary) messages (includes questions on readiness to start exercising with/out consulting a physician first), some ideas to be active, some techniques to cope with challenges, and some ways to stay motivated to favour and facilitate PA practice.

The booklet supplements/compliments PA educational session content (within the SA-DPP intervention curriculum) which was similarly developed, based on population needs and context as well as relevant literature and expert recommendations. The SA-DPP curriculum includes information on PA and its benefits in general, intensity, and required frequency. A practical demonstration session was also added, based on the exercises proposed in the booklet.

### 4.2. Overcoming the Barriers to PA

Several barriers to PA were identified in the reviewed papers. Laziness (or lack of motivation) [33,42], fatigue [36,44], and lack of money [46] appeared to be the most common ones in the reviewed studies. In the current study, similarly overcoming laziness, a lack of facilities and lack of money to access gyms were highlighted as barriers. Two main strategies were identified to overcoming laziness, i.e., the importance of friends and familial support [33] and the organisation of events in communities [42]. These are plausible strategies within our study population, as the FGDs emphasised social support and the initiation of community walking groups and aerobic classes along with existing ones. Fatigue may result from a programme that may be too intensive and lead to dropout. Therefore, the intensity, volume and duration of exercise must be balanced and pragmatic. The capacity and context of the study population must be taken into consideration to limit the number of dropouts [36,44]. Gym membership and people’s involvement in PA increased when membership fees decreased and when they were rewarded for participating in fitness activities. Thus, finance is involved in a participant’s daily life choices, and the role of incentives seemed significant, as it could lower the motivational and financial barriers [46].

## 5. Conclusions

The PA booklet that was developed to suit the context-specific needs of the population within the SA-DPP study, will likely enable this study population to increase their moderate-intensity PA practices at home, without the need for additional resources. This population is at risk for developing T2DM. Therefore, lifestyle changes, especially regarding PA and nutrition, can delay the onset of T2DM and improve their overall health and quality of life.

## Figures and Tables

**Table 1:** Measurement domains, tools, and data collection for the South African Diabetes Prevention Programme’s pilot phase.

Variable	Component	Measurements Tools/Questions
**Socio-demographic measures**		Age, gender, area, community, current marital status, education level, occupation, income
**Behavioural measures**	Tobacco use	WHO STEPS questionnaire [14]
	Alcohol use	WHO STEPS questionnaire [14]
	Sedentary behaviour	Time spent in front of a screen
	Sleep	Time, quality
**Psychological measures**	Chronic stress	Chronic stress scale [13]
**Physical activity measures**	Physical activity pattern	WHO STEPS questionnaire: global physical activity questionnaire (GPAQ) [14]
	Barriers to physical activity	Scale adapted from the one designed by Booth et al. [15]
	Self-efficacy	Scale adapted from the exercise self-efficacy scale (ESES) designed by Schwarzer and Jerusalem [16]
**Medical history**		Family history of diabetes
**Clinical measures**	Waist circumference	Measured between the lower border of the lowest rib and upper border of the iliac crest/pelvic bone to the nearest 0.1 cm.
	Weight	Weight measurement with minimal clothing on a digital (SECA) scale, recorded to the nearest 0.1 kg
	Height	Standing height, minimal clothing, aligning head in a standard anatomical position using a SECA stadiometer
	SBP	Electronic M6 COMFORT OMRON device with an integrated cuff
	DBP HbA1c	Electronic M6 COMFORT OMRON device with an integrated cuff HbA1c measured using fasting blood and HPLC
**Neighbourhood indicators**	Stores and facilities, Access to services and places, Roads and walking paths, places for walking/cycling/playing, Surroundings, Safety from crime and traffic, Personal safety, Stranger danger	Neighbourhood Environment Walkability Scale (NEWS) Africa Questionnaire [17]

SBP = systolic blood pressure; DBP = diastolic blood pressure; HbA1c = glycosylated haemoglobin; HPLC = High-performance liquid chromatography; WHO = World Health Organisation.

**Table 2:** Socio-demographic characteristics of at-risk type II diabetes black and mixed-ancestry communities in Cape Town, 2018 (N = 316).

Socio-Demographic Characteristics (N = 316)		
	N	%
**Age, median (25th–75th percentiles)**	53.00 [46.00–59.00]	
25–45 years	57	18.0
45–65 years	256	**81.0**
Not Assigned	3	1.0
**Gender**		
Male	60	19.0
Female	253	**80.1**
NA	3	0.9
**Community**		
Black	172	**54.4**
Mixed-ancestry	141	44.6
Not Assigned	3	1.0
**Education**		
Never went to school	2	0.6
Primary school (Grades 1–7)	76	24.1
High school (Grades 8–12)	136	**43.0**
Less than Grade 12 + FET */Certificate/Diploma	11	3.5
Grade 12 and higher (Tertiary/Diploma/Degree)	86	27.2
NA	5	1.6
**Occupation**		
Employed (full- or part-time/self-employed/	92	29.1
Unemployed	123	**38.9**
Full-time homemaker	21	6.6
Pensioner	58	18.4
On a disability grant	13	4.1
Child grant	4	1.3
Not Assigned	5	1.6
**Income**		
No income	32	10.1
R1–R800	39	12.3
R801–R1,600	42	23.4
R1601–R3200	94	**29.8**
R3201–R6400	74	13.3
R6401–R12,800	19	6.0
R12,801–R51,200	11	3.5
Not Assigned	5	1.6

* FET—Further Education and Training.

**Table 3:** Biological and behavioural risk factors for type 2 diabetes among at-risk black and mixed-ancestry communities in Cape Town, 2018 (N = 316).

Risks Factors (N = 316)		
	N	%
**Waist circumference (cm), median (25th–75th percentiles) *(NA = 4)***	102.00 [95.08–111.11]	
**Body mass index (BMI, kg/m^2^), median (25th–75th percentiles)**	34.85 [29.93–40.74]	
**BMI**		
Underweight (<18.5)	0	0.0
Normal weight (18.5–24.9)	12	3.8
Overweight (25.0 to 29.9)	67	21.2
Obese	233	**73.7**
Class 1 (30.0 to 34.9)	80	34.3
Class 2 (35.0 to 39.9)	62	26.6
Class 3 (≥40)	91	**39.1**
Not documented	4	1.3
**Family medical history**		
Having at least one known diabetic close relative	141	**44.6**
Do not have one known diabetic close relative	57	18.0
Do not know	114	36.1
Not documented	4	1.3
**Blood pressure**		
Optimal/normal (<120/120–129 mmHg/<80/80–84 mmHg)	129	**40.8**
High normal (130–139 mmHg/85–89 mmHg)	71	22.5
Hypertension (≥140 mmHg/90 mmHg)	93	29.4
Isolated systolic hypertension (≥140 mmHg/<90 mmHg)	19	6.0
Not documented	4	1.3
**Glycated haemoglobin (HbA1c)**		
<5.7 mmol/L	99	31.3
≥5.7 mmol/L	206	**65.2**
Not documented	11	3.5
**Alcohol consumption**		
Abstainer	174	**55.1**
Less than once a month	51	16.1
1–3 days per month	45	14.2
Several times per week	42	13.3
Not documented	4	1.3
**Tobacco status**		
Non-smoker (never smoked tobacco)	190	**60.1**
Current Smoker (daily or occasionally)	83	26.3
Ex-smoker	40	12.7
Not documented	3	0.9
**Stress**		
Having an ongoing problem/stressor	154	**48.7**
Having no chronic difficulties	157	49.7
Not documented	5	1.6

**Table 4:** Self-reported physical activity (PA) pattern (GPAQ questionnaire) among at-risk type 2 diabetes black and mixed-ancestry communities in Cape Town, 2018 (N = 305).

Physical Activity Patterns	Median	25th–75th Percentiles
**Total time spending doing PA (minutes/week)**	720.00	[240.00–1710.00]
At work	360.00	[0.00–1050.00]
For transport	180.00	[60.00–360.00]
For leisure	30.00	[0.00–180.00]
**Sedentary (minutes/week)**	1260.00	[630.00–1680.00]
	N	%
**Practice of PA**		
**In general**		
Yes	290	**95.1**
No	15	4.9
**At work**		
Yes	221	**72.5**
No	84	27.5
**For transport**		
Yes	265	**86.9**
No	40	13.1
**For leisure**		
Yes	157	**51.5**
No	148	48.5
**Vigorous-intensity**		
Yes	39	12.8
No	266	**87.2**
WHO recommendations *		
Does not meet recommendations	40	13.1
Meets recommendations	265	**86.9**

* World Health Organisation (WHO) PA recommendations (150 min of moderate activity or 75 min of vigorous activity per week).

**Table 5:** Limitations in daily activities attributable to physical health problems among at-risk type 2 diabetes black and mixed-ancestry communities, Cape Town, 2018 (N = 316).

Short Form-36—Physical Functioning Subscale		
	N	%
**Poor health limits the following activities:**		
Vigorous activities	202	**64.3**
Moderate activities	101	**32.2**
Lift/carry groceries	62	19.7
Climb several flights of stairs	104	**33.1**
Climb one flight of stairs	74	23.6
Bending/kneeling/stooping	99	**31.5**
Walk more than one kilometre	75	23.9
Walk several hundreds of meters	82	26.1
Walk one hundred meters	73	23.2
Bath and dress oneself	23	7.3

## Appendix A

Final FGD code list.

## Appendix B

Literature review: strategy, overview and results.

## Appendix C

Physical activity booklet.

## References

[1] International Diabetes Federation (2017). IDF Diabetes Atlas Eighth Edition.

[2] Erasmus R.T., Soita D.J., Hassan M.S., Blanco-Blanco E., Vergotine Z., Kengne A.P., Matsha T.E. (2012). High prevalence of diabetes mellitus and metabolic syndrome in a South African coloured population: Baseline data of a study in Bellville, Cape Town. S. Afr. Med. J..

[3] Peer N., Steyn K., Lombard C., Lambert E.V., Vythilingum B., Levitt N.S. (2012). Rising Diabetes Prevalence among Urban-Dwelling Black South Africans. PLoS ONE.

[4] Kruger H.S., Puoane T., Senekal M., Van der Merwe M.T. (2005). Obesity in South Africa: Challenges for government and health professionals. Public Health Nutr..

[5] Jefferis B.J., Whincup P.H., Lennon L., Wannamethee S.G. (2012). Longitudinal Associations Between Changes in Physical Activity and Onset of Type 2 Diabetes in Older British Men. Diabetes Care..

[6] Boyle T., Keegel T., Bull F., Heyworth J., Fritschi L. (2012). Physical activity and risks of proximal and distal colon cancers: A systematic review and meta-analysis. J. Natl. Cancer Inst..

[7] Sattelmair J., Pertman J., Ding E.L., Kohl H.W., Haskell W., Lee I.M. (2011). Dose response between physical activity and risk of coronary heart disease: A meta-analysis. Circulation.

[8] Lee C.D., Folsom A.R., Blair S.N. (2003). Physical activity and stroke risk: A meta-analysis. Stroke.

[9] Tanasescu M., Leitzmann M.F., Rimm E.B., Willett W.C., Stampfer M.J., Hu F.B. (2002). Exercise type and intensity in relation to coronary heart disease in men. JAMA.

[10] Lindstrom J., Louheranta A., Mannelin M., Rastas M., Salminen V., Eriksson J., Uusitupa M., Tuomilehto J., Finnish Diabetes Prevention Study Group (2003). The Finnish Diabetes Prevention Study (DPS): Lifestyle intervention and 3-year results on diet and physical activity. Diabetes Care.

[11] Knowler W.C., Barrett-Connor E., Fowler S.E., Hamman R.F., Lachin J.M., Walker E.A., Nathan D.M., Diabetes Prevention Program Research Group (2002). Reduction in the incidence of type 2 diabetes with lifestyle intervention or metformin. N. Engl. J. Med..

[12] Absetz P., Valve R., Oldenburg B., Heinonen H., Nissinen A., Fogelholm M., Ilvesmäki V., Talja M., Uutela A. (2007). Type 2 diabetes prevention in the “real world”: One-year results of the GOAL Implementation Trial. Diabetes Care..

[13] Bromberger J.T., Matthews K.A. (1996). A longitudinal study of the effects of pessimism, trait anxiety, and life stress on depressive symptoms in middle-aged women. Psychol. Aging.

[14] World Health Organization (2018). Global Physical Activity Questionnaire (GPAQ).

[15] Booth M.L., Bauman A., Owen N., Gore C.J. (1997). Physical activity preferences, preferred sources of assistance, and perceived barriers to increased activity among physically inactive Australians. Prev. Med..

[16] Schwarzer R., Jerusalem M., Weinman J., Johnston M. (1995). Generalized Self-Efficacy scale. Measures in Health Psychology: A User’s Portfolio Causal and Control Beliefs Windsor.

[17] Oyeyemi A.L., Kasoma S.S., Onywera V.O., Assah F., Adedoyin R.A., Conway T.L., Moss S.J., Ocansey R., Kolbe-Alexander T.L., Akinroye K.K. (2016). NEWS for Africa: Adaptation and reliability of a built environment questionnaire for physical activity in seven African countries. Int. J. Behav. Nutr. Phys. Act..

[18] Jepson R., Harris F.M., Bowes A., Robertson R., Avan G., Sheikh A. (2012). Physical activity in South Asians: An in-depth qualitative study to explore motivations and facilitators. PLoS ONE.

[19] Miller S.T., Marolen K. (2012). Physical activity-related experiences, counseling expectations, personal responsibility, and altruism among urban African American women with type 2 diabetes. Diabetes Educ..

[20] Pope C., Ziebland S., Mays N. (2000). Qualitative research in health care. Analysing qualitative data. Br. Med J..

[21] Babbie E.R., Mouton J. (2001). The Practice of Social Research.

[22] Zlatkovic M., Zlatkovic S., Sullivan T., Bjornstad J., Kiavash F.S.S. (2019). Assessment of effects of street connectivity on traffic performance and sustainability within communities and neighborhoods through traffic simulation. Sustain. Cities Soc..

[23] Colberg S.R., Sigal R.J., Fernhall B., Regensteiner J.G., Blissmer B.J., Rubin R.R., Chasan-Taber L., Albright A.L., Braun B., American College of Sports Medicine (2010). Exercise and type 2 diabetes: The American College of Sports Medicine and the American Diabetes Association: Joint position statement. Diabetes Care.

[24] Irvine C., Taylor N.F. (2009). Progressive resistance exercise improves glycaemic control in people with type 2 diabetes mellitus: A systematic review. Aust. J. Physiother..

[25] Holten M.K., Zacho M., Gaster M., Juel C., Wojtaszewski J.F., Dela F. (2004). Strength training increases insulin-mediated glucose uptake, GLUT4 content, and insulin signaling in skeletal muscle in patients with type 2 diabetes. Diabetes.

[26] Sigal R.J., Kenny G.P., Wasserman D.H., Castaneda-Sceppa C., White R.D. (2006). Physical activity/exercise and type 2 diabetes: A consensus statement from the American Diabetes Association. Diabetes Care.

[27] Lesinski M., Hortobagyi T., Muehlbauer T., Gollhofer A., Granacher U. (2015). Effects of Balance Training on Balance Performance in Healthy Older Adults: A Systematic Review and Meta-analysis. Sports Med..

[28] Westcott W.L. (2012). Resistance training is medicine: Effects of strength training on health. Curr. Sports Med. Rep..

[29] Shaw B.S., Shaw I., Brown G.A. (2015). Resistance exercise is medicine: Strength training in health promotion and rehabilitation. Int. J. Ther. Rehabil..

[30] Onagbiye S.O., Moss S.J., Cameron M. (2016). Managing Noncommunicable Diseases in an African Community: Effects, Compliance, and Barriers to Participation in a 4-Week Exercise Intervention. Int. Q. Community Health Educ..

[31] Mathunjwa M.L., Semple S.J., du Preez C. (2013). A 10-week aerobic exercise program reduces cardiometabolic disease risk in overweight/obese female African university students. Ethn. Dis..

[32] Jemmott J.B., Jemmott L.S., Ngwane Z., Zhang J., Heeren G.A., Icard L.D., O’Leary A., Mtose X., Teitelman A., Carty C. (2014). Theory-based behavioral intervention increases self-reported physical activity in South African men: A cluster-randomized controlled trial. Prev. Med..

[33] Roos R., Myezwa H., Van Aswegen H. (2015). “Not easy at all but I am trying”: Barriers and facilitators to physical activity in a South African cohort of people living with HIV participating in a home-based pedometer walking programme. AIDS Care..

[34] Chetty L., Ramklass S.S., McKUNE A.J. (2014). The Effects of a Structured Group Exercise Programme on Functional Fitness of Older Persons Living in Old-Age Homes. Master’s Thesis.

[35] Pillay J.D., Van der Ploeg H.P., Kolbe-Alexander T.L., Proper K.I., Van Stralen M., Tomaz S.A., van Mechelen W., Lambert E.V. (2015). The association between daily steps and health, and the mediating role of body composition: A pedometer-based, cross-sectional study in an employed South African population. BMC Public Health.

[36] Ley C., Leach L., Barrio M.R., Bassett S. (2014). Effects of an exercise programme with people living with HIV: Research in a disadvantaged setting. Afr. J. AIDS Res. AJAR.

[37] Owen N., Healy G.N., Matthews C.E., Dunstan D.W. (2010). Too much sitting: The population health science of sedentary behavior. Exerc. Sport Sci. Rev..

[38] Dunstan D.W., Salmon J., Healy G.N., Shaw J.E., Jolley D., Zimmet P.Z., Owen N., On behalf of the AusDiab Steering Committee (2007). Association of television viewing with fasting and 2-h postchallenge plasma glucose levels in adults without diagnosed diabetes. Diabetes Care.

[39] Yang Z., Scott C.A., Mao C., Tang J., Farmer A.J. (2014). Resistance exercise versus aerobic exercise for type 2 diabetes: A systematic review and meta-analysis. Sports Med..

[40] Jorge M.L., De Oliveira V.N., Resende N.M., Paraiso L.F., Calixto A., Diniz A.L., Resende E.S., Ropelle E.R., Carvalheira J.B., Espindola F.S. (2011). The effects of aerobic, resistance, and combined exercise on metabolic control, inflammatory markers, adipocytokines, and muscle insulin signaling in patients with type 2 diabetes mellitus. Metab. Clin. Exp..

[41] Wallberg-Henriksson H., Rincon J., Zierath J.R. (1998). Exercise in the management of non-insulin-dependent diabetes mellitus. Sports Med..

[42] Skaal L., Pengpid S. (2012). The predictive validity and effects of using the transtheoretical model to increase the physical activity of healthcare workers in a public hospital in South Africa. Transl. Behav. Med..

[43] Mash B., Levitt N., Steyn K., Zwarenstein M., Rollnick S. (2012). Effectiveness of a group diabetes education programme in underserved communities in South Africa: Pragmatic cluster randomized control trial. BMC Fam. Pract..

[44] Van der Does A.M., Mash R. (2013). Evaluation of the “Take Five School”: An education programme for people with Type 2 Diabetes in the Western Cape, South Africa. Prim. Care Diabetes.

[45] Plow M., Bethoux F., Mai K., Marcus B. (2014). A formative evaluation of customized pamphlets to promote physical activity and symptom self-management in women with multiple sclerosis. Health Educ. Res..

[46] Patel D., Lambert E.V., Da Silva R., Greyling M., Kolbe-Alexander T., Noach A., Conradie J., Nossel C., Borresen J., Gaziano T. (2011). Participation in fitness-related activities of an incentive-based health promotion program and hospital costs: A retrospective longitudinal study. Am. J. Health Promot..

